# Hair follicle stem cells promote epidermal regeneration under expanded condition

**DOI:** 10.3389/fphys.2024.1306011

**Published:** 2024-02-22

**Authors:** Yu Zhang, Jiangbo Cui, Zhengqiang Cang, Jiaomiao Pei, Xi Zhang, Baoqiang Song, Xing Fan, Xianjie Ma, Yang Li

**Affiliations:** Department of Plastic Surgery, Xijing Hospital, Fourth Military Medical University, Xi’an, Shaanxi, China

**Keywords:** hair follicle stem cells, epidermal regeneration, skin soft tissue expansion, mechanical stretch, wound healing

## Abstract

Skin soft tissue expansion is the process of obtaining excess skin mixed with skin development, wound healing, and mechanical stretching. Previous studies have reported that tissue expansion significantly induces epidermal proliferation throughout the skin. However, the mechanisms underlying epidermal regeneration during skin soft tissue expansion are yet to be clarified. Hair follicle stem cells (HFSCs) have been recognized as a promising approach for epidermal regeneration. This study examines HFSC-related epidermal regeneration mechanisms under expanded condition and proposes a potential method for its cellular and molecular regulation.

## 1 Introduction

Skin soft tissue expansion plays a crucial role in plastic and reconstructive surgery, and is widely used for organ reconstruction, repair of extensive scarring, giant congenital nevi and tissue defects (Cheng et al., 2020). During the expansion, the expander implanted subcutaneously is continuously inflated with regular saline injections, thereby resulting in the acquisition of skin tissues that are adequately to cover the wound defects matched in color and texture. Despite the safety and satisfactory repair effects of skin soft tissue expansion, a serious problem persists which is the low expansion efficiency (Dong et al., 2020), prolonging the treatment duration for patients (Han et al., 2017). To addressed this problem, facilitating skin regeneration becomes a promising approach which links to series of underlying mechanisms (Guo et al., 2022).

Skin tissues undergo physiological growth primarily through biological responses to mechanical stretch, as they are subjected to forces from the inflation of the expander beneath the skin during the whole period of skin expansion over months. Therefore, mechanical stretching is increasingly regarded as the major and initiated factor influencing skin regeneration. During expansion, skin tissues undergo repeated microtrauma, wound healing and skin development in response to mechanical stretch stimulation (Ding et al., 2019). In such expanded condition, complex mechanobiological microenvironment induces skin tissues sensing the stretching accompanied by cellular and extracellular matrix reshaping (Guimarães et al., 2020), thereby resulting in multiple biological reactions different from simple wound healing and skin renewal. Consequently, various cells, molecules, and signaling pathways undergo constant changes, thereby leading to the generation of new tissues. Cell behaviors and fates are determined by a multitude of differentially expressed genes (DEGs), which can be influenced by mechanical stimulation and subsequently affect gene expressions. Additionally, mechanics-related signaling pathways (Misra and Irvine, 2018) play crucial roles in this process.

Recently, significant variations in the skin were reported including the epidermis, dermis, and cutaneous tissues under expanded condition (Yu et al., 2020; Zhang and Beachy, 2021). The most noticeable changes throughout the skin were observed in the epidermis (Huang et al., 2023), in which hair follicle (HF) exhibits vigorous hair growth (Lee et al., 2019). Studies have shown that the hair follicle stem cells (HFSCs) participate in tissue repair (Li et al., 2022; Sun et al., 2023). HFSCs are located in the bulge region of the HF, which is spatially relevant to the skin (Joulai et al., 2017). HFSCs have recently gained significant attention for their role in skin repair (Li et al., 2019) because they can differentiate into other cells (Quan et al., 2017; Schomann et al., 2020). In addition to having a greater potential for diverse differentiation, HFSCs are easier to obtain with less harm to donors than other stem cells (Wang et al., 2013; Chen and Guan, 2023). Though HFSCs are reported to make a nonnegligible contribution to epidermal regeneration under expanded condition (Cheng et al., 2020), the underlying mechanisms still have not been thoroughly clarified.

This review elucidates the cell crosstalk and molecular mechanisms (transcriptome changes, signaling pathways, and ion channels) of HFSCs under expanded condition, thereby contributing to a deeper understanding of HFSCs-dependent epidermal regeneration.

## 2 HFSCs in epidermal regeneration

### 2.1 HFs and HFSCs

**FIGURE 1:**
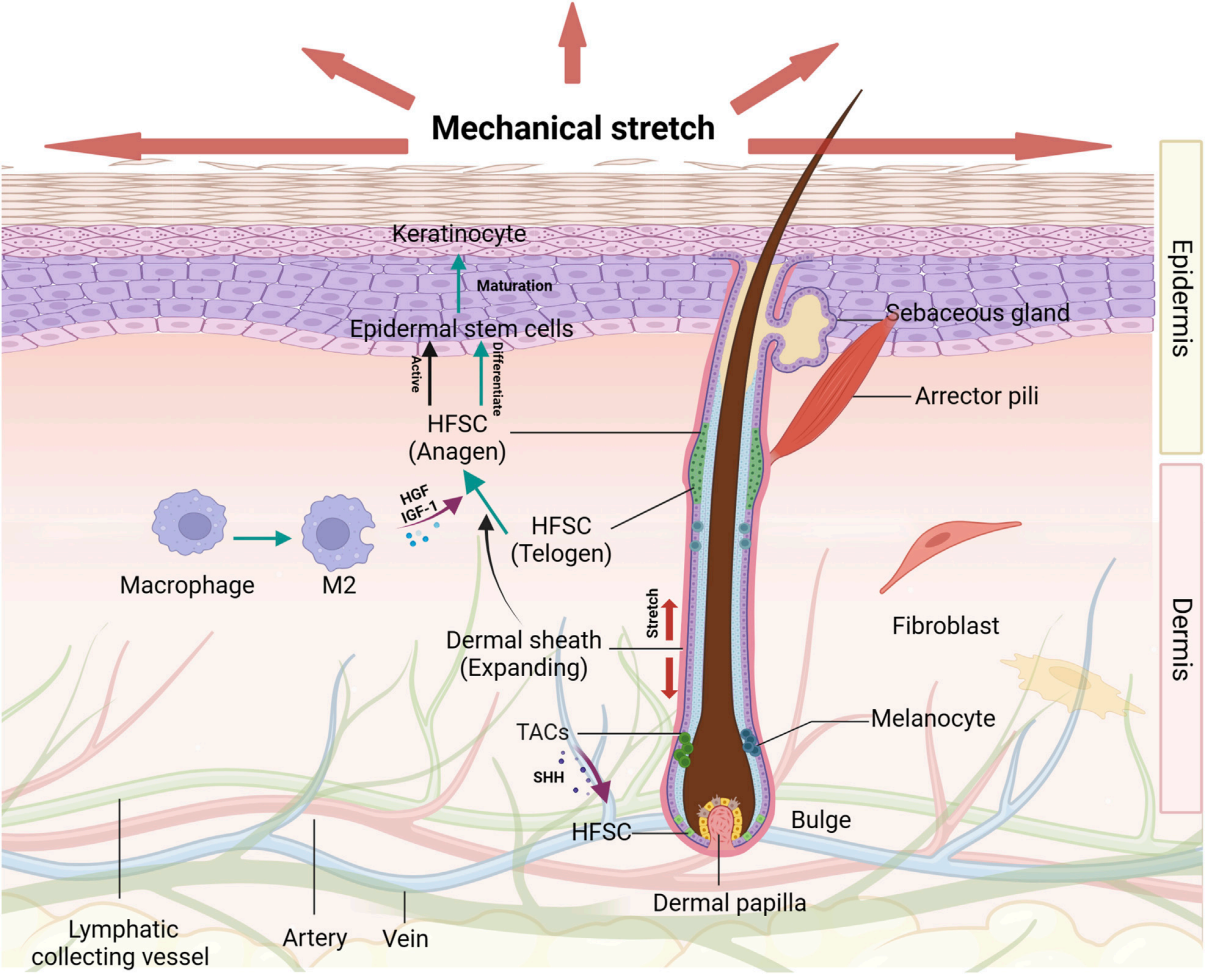
The effects of mechanical stretch on HFSCs and their niches Diverse groups of cells and extracellular matrix proteins around the HFSCs are organized to form a niche that serves to promote and maintain the optimal functioning of these stem cell populations. The hair cycle and HFSCs activation condition are changed induced by mechanical stretch. Under certain circumstances, HFSCs are transformed into epidermal stem cells which can be promoted by several growth factors secreted by M2 macrophages. These effects on HFSCs play important roles in epidermal regeneration.

In mammalian skin, hair and hair follicles (HFs) are appendages crucial for protection, thermal regulation, and sensory perception. Throughout the life, HFs experience repetitive regenerative cycles that produce hair propelled by the intermittent activation of HFSCs (Hu et al., 2021; Lee et al., 2023). HFs, consisting of the papilla, matrix, dermal sheath, and bulge, are complex mini-organs embedded in the skin. The dermal sheath contains the bulge region, which is the major repository of HFSCs. It provides a microenvironment, known as the “niche”, which is pivotal in supporting functions of these stem cell groups and regulating successive hair growth cycles, including telogen, anagen, and catagen phases. The skin stem cells and their niches are shown in Figure 1. HFSCs exhibit heterogeneity, with those in the upper bulge not contributing to HF regeneration, also called isthmic stem cells, whereas those in the lower bulge can regenerate the outer root sheath of the HF (Hu et al., 2021). During HF morphogenesis, the lower portion undergoes repetitive degeneration and regeneration, which is contributed by HFSCs (Hsu et al., 2014).

**FIGURE 2:**
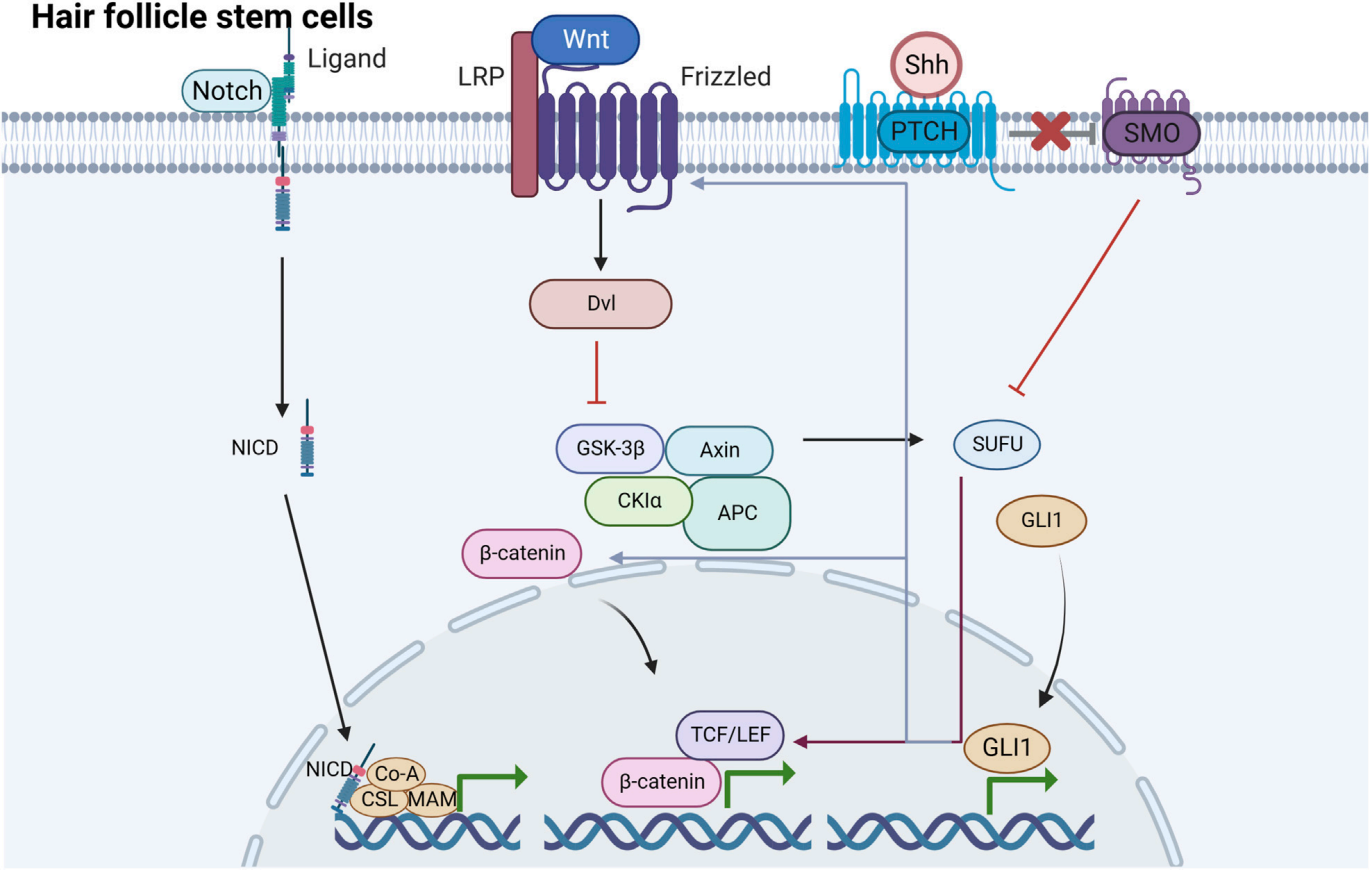
Signaling pathways induced by HFSCs under mechanical stretching There are mainly three signaling pathways induced by HFSCs under mechanical stretching including Wnt/β-catenin, Shh and Notch pathway. They are underlying mechanisms of HFSCs promoting epidermal regeneration.

Isthmic stem cells are distinguished at the upper region of the HF by low α6-integrin levels and are negative for CD34. Research has identified a versatile population of HFSCs are situated close to the sebaceous gland. These cells are referred to HF-associated pluripotent stem cells, and their offspring are located in the outer root sheath of the HF (Li et al., 2023; Amoh and Hoffman, 2017). Stem cells in the upper bulge area, usually dormant but with self-renewal capabilities, are the primary source for the upper hair and sebaceous unit (Rompolas et al., 2013). Han et al. reported that the descendants of K15^+^, Lgr5^+^, and Gli1^+^ stem cells from the upper bulge region contribute to the formation of sebocytes through a process reliant on β-catenin (Han et al., 2017). Additionally, these cells can differentiate into various cell types, including neuronal, endothelial, and fat cells, beyond the HF (Hoffman, 2006; Amoh et al., 2005; Amoh and Hoffman, 2017). Figure 2 Similarly, these cells also exhibit significant clonogenic, multipotent, and self-renewal capabilities and contribute to wound healing (Lough et al., 2016).

HFSCs are located in the bulge and remain quiescent in the telogen phase. HFSCs may experience activation–quiescence transitions depending on their niche microenvironment, such as the cycling of HFs and tissue injury. The status of HFSCs relies on the factors originated from the microenvironment. When a certain stem cell activator concentration is reached, some HFSCs will be activated, accompanied by the beginning of the anagen phase (Ji et al., 2021) while the remaining HFSCs still maintained quiescence. Activated HFSCs play a role in hair growth and tissue regeneration, exhibiting versatility in response to various external stimuli (Zhang et al., 2023; Shwartz et al., 2020). Studies have highlighted the significance of the microenvironment, especially the role of local mechanics in tissue engineering and regeneration. Additionally, cells adjacent to HFSCs in their niche modulate HFSC activity via secreted molecules such as fibroblast growth factors and bone morphogenetic protein inhibitors or direct interactions between cells (Rendl et al., 2008).

Increasingly, HFSCs are recognized as vital reservoirs for epidermal tissue regeneration post-injury. In the 1940s, researchers first reported that dedifferentiated cells from HFs contribute to wound healing, and superficial wounds where HFs remained intact healed faster (Toma et al., 2021). Moreover, grafts containing HFs carrying HFSCs have shown great potential in promoting epidermal healing for the treatment of chronic cutaneous wounds (Liu et al., 2015; Martínez et al., 2016; Martínez et al., 2017). HFSCs in the lower bulge region and the isthmus of the HF both can migrate to the injury sites and contribute to re-epithelialization during wound healing (Nuutila et al., 2017; Burgy and Königshoff, 2018; Li et al., 2019). Cells located in the HF lower bulge have the capability to generate both the HF and epidermis, especially in injured newborn mouse skin. In these scenarios, cells within the HF bulge can migrate towards the epidermis (Liu et al., 2024). Recently, a study showed that when a superficial wound was created in mice, the HF cells in the newly formed epidermis were gradually replaced by keratinocytes derived from the epidermis (Ji et al., 2021). It is indicated that HFSCs in the bulge region predominantly react to epidermal wounds by generating short-lived “transient amplifying” cells, which play a part in the recovery of acute wounds (Lee and Choi, 2024). Langton et al. reported that when a wound was created in the tail of mutant mice lacking all HF development, the cutaneous wounds healed with a delay re-epithelialization (Langton et al., 2008).

Therefore, the ability of HFSCs to differentiate and regenerate is crucial for epidermal regeneration; however, the mechanisms involved are intricate and worth exploring.

### 2.2 The crosstalk between HFSCs and other cells in epidermal regeneration

The epidermis, serving as the primary environmental barrier, adjusts its form and dimensions in response to mechanical stretching (Khorasani and Sadeghi, 2024). HFSCs promote epidermal regeneration caused by the connections between HFSCs and cells including keratinocytes, endothelial cells (ECs), and inflammatory cells (Gonzales and Fuchs, 2017), which are of great help in epidermal regeneration. Furthermore, there exists conversion such as differentiation in cell types in response to mechanical stretching, which has tight relationship with HF regeneration and wound healing (Rompolas et al., 2013). Conversely, the complex interactions among different cells form a feedback loop through growth factors or overlapping signaling pathways that regulate the functions of each cell (Hsu et al., 2014).

#### 2.2.1 HFSCs and keratinocytes

Keratinocytes constitute the epidermis and serve as a skin barrier against external damage. Skin typically undergoes epithelialization when damaged by outside stimuli. During this process, keratinocytes are derived from the interfollicular epidermal stem cells in the basal layer as well as from HFSCs and their own proliferation. Epidermal stem cells reside in the basal layer of the epidermis and exhibit limited division. They are also accountable for differentiation into different lineages within mature skin (Yang et al., 2020). The main site of epidermal stem cell regeneration is the stratified epidermis, which forms a physical barrier. Epidermal stem cells undergo either symmetric division yielding two same stem cells, or asymmetric division generating progenitor cells and ‘reserve’ non-stem cells with slow cycling properties. The slow cycling stem cells are favored of its turnover which are crucial for tissue regeneration. Lineage tracing reveals that slow cycling cells, once mobilized, generate cell clones that contribute to healing and then the healing areas are characterized by a self-renewing interfollicular epidermis. Despite their minimal role in tissue homeostasis, these cells are mobilized post-injury, significantly aiding epidermal repair in areas devoid of HFs. It is notable that in most cases, symmetric divisions are characterized by a prominent population of self-renewal rather than exclusive asymmetric division.

Daughter cells that have differentiated move away from the basal layer and undergo further differentiation in the spinous, granular, and suprabasal layers of the stratum corneum. Inward-moving inner cells eventually replace these cells (Hsu and Fuchs, 2022). Hence, the importance of epidermal stem cells lies in their ability to repair skin defects, restore skin integrity, enhance tensile strength, and improve barrier function (Singer et al., 1999). During wound healing and skin regeneration, epidermal stem cells acquire the ability to repair adjacent compartments, and these compartments can be refilled with each other (Sada et al., 2016; Hirsch et al., 2017; Lee and Choi, 2024). Observed that mechanical stretching and epidermal stem cells are closely correlated and regulated by EZH2 (Wang et al., 2021; Wang et al., 2021).

HFSCs and epidermal stem cells can undergo mutual transformation under certain conditions. They play a crucial role in wound healing by recruiting and differentiating into epidermal cells, a process that can be facilitated by exogenous cytokines, growth factors, and chemicals (Matsumura et al., 2021). Gli1^+^ HFSCs are more prone to differentiate into epidermal stem cells (Lee et al., 2023). As HFSCs and epidermal stem cells are responsive to mechanical traction and influenced by various factors, whether endogenous or exogenous, a deeper understanding of the underlying mechanisms is crucial. Therefore, the transition of HFSCs to keratinocytes may be achieved via epidermal stem cells under expanded condition.

#### 2.2.2 HFSCs and ECs

It is important for cellular regeneration and tissue repairing to obtain an accelerated neovascularization, increasing blood flow and nutrition in surroundings. Cheng et al. confirmed that during skin expansion, HFSCs can differentiate into vascular ECs stimulated by the secretion of growth factors, including EGF, VEGF, bFGF, and TGF-β (Cheng et al., 2020). After incubating HFSCs with VEGF and bFGF for 7 days, Xu *et al.* found that the expanded HFSCs displayed characteristics similar to endothelial cells, such as expressing vWF, VE-cadherin, and CD31 (Xu et al., 2014). Although the interplay of HFSCs and ECs has less been reported under expanded condition, they indeed play a crucial role in epidermal regeneration because tissue regeneration deeply relies on neovascularization and re-epithelization. For example, in angiogenesis, which is vital for skin barrier rebuilding (Liu et al., 2024), Heidari reported that HF bulge cells increased vascularization in full-thickness wounds in mice instead of HF isthmus cells (Heidari et al., 2016). In the study conducted by Babakhani, when HFSCs were administered around the periphery of the wound, it was observed that the wound area exhibited significant enhanced neovascularization, particularly notable on the 7th and 14th days following treatments, in comparison to other groups (Babakhani et al., 2020). These newly formed blood vessels were instrumental in reducing the duration of the inflammatory phase and in supplying essential nutrients and oxygen for the proliferation of cells and the regeneration of tissues (Xia et al., 2017). The effects of HFSCs on vascularization may be due to the differentiation of HFSCs to ECs, which may also be closely associated with the factors HFSCs promote to secrete.

#### 2.2.3 HFSCs and inflammatory cells

Inflammatory cells, including macrophages and regulatory T cells, promote quiescence and hair growth in HFSCs, leading to epidermal regeneration.

Typically, there is a notable number of macrophages found in the perifollicular compartment. They regulate hair cycles and enable the HFSC niche to detect tissue injury and mechanical states. Initially, they stimulate HFSC activation and differentiation by releasing factors like Wnt7b and Wnt10a. Additionally, the depletion of TREM2 dermal macrophages, producers of oncostatin M, inhibits downstream JAK-STAT signaling resulting in premature anagen phase entry. During wound healing, macrophages are recruited via apoptosis signal-regulating kinase 1 (ASK1) or CCL2, activating HFSCs through the TNF-α/AKT/β-catenin pathway. M2 macrophages produced in stretched skin facilitate hair regeneration by secreting growth factors such as IGF and HGF (Chu et al., 2019). This illustrates that various populations of macrophages play separate roles in regulating HFSC activation and differentiation.

In the skin, regulatory T cells (Tregs) primarily localize to HFs, maintaining the HF niche. Numbers of Tregs are mainly found surrounding HFs during the resting phase of the HF cycle. They not only function traditionally as an inflammation controller, promoting tissue homeostasis, but enhance the activation and differentiation of HFSCs, promoting HF cycling. Tregs were proved to facilitate the HF telogen-to-anagen transition accompanied by the increasing number of proliferating Ki67 HFSCs which was significantly lower in Treg depleted mice. Under the condition of wound healing especially after epidermal injury, HFSCs are recruited from the HF bulge and participate in the repair of the upper HF and interfollicular epidermis through controlling the specific IL17-CXCL5-neutrophil axis (Lee et al., 2023). However, the inhibition of inflammation is not the only way in which Tregs promote HFSCs function in the process of hair regeneration. In certain research studies, Tregs produce the Notch ligand, Jag1, which stimulates the proliferation and differentiation of HFSCs and drives progression through the anagen phase (Ali et al., 2017). HFSCs behaviors are changed during HF cycling through Notch signal which is employed by skin-resident Tregs. Interestingly, it is also found that the glucocorticoid receptor (GR) in Treg cells affects hair regeneration without disturbing immune homeostasis. GR promoted TGF-β3 induction in Treg cells which activates Smad2/3 in HFSCs and facilitates HFSC proliferation (Liu et al., 2022).

Interestingly, some researchers have also discovered that HFSCs acquire an “inflammatory memory” (Cheng et al., 2023). It is well-known that only certain immune cells develop memory to shield tissues from external stimuli. However, Fuchs *et al.* demonstrated that upon skin inflammation or trauma, HFSCs resist stimuli, proliferate, and differentiate, thereby the damaged epidermal cells are subsequently replaced (Naik et al., 2017). Even if the stimuli disappeared, some cells maintained a post-inflammatory characteristic for a long time, speeding up the wound healing process. It may be related to chromatin remodeling and enhanced inflammation-related transcriptional response, augmenting IL-1β to promote the regenerative process. At present, the inflammatory memory state in HFSCs could persist for at least 180 days, showing the stability of the response.

## 3 Mechanisms of epidermal regeneration induced by HFSCs under mechanical stretching

The mechanisms of epidermal regeneration are part of a comprehensive and complex system, as HFSCs are involved in transcriptional, molecular, and signaling pathway regulation under mechanical stretching.

### 3.1 Transcriptome changes

Recently, to elucidate gene information under various microenvironments, transcriptomic analysis using RNA sequencing has been increasingly employed, reflecting the fundamental differential expression of RNA levels. To examine the underlying molecular mechanisms, several researchers have endeavored to investigate transcriptomic changes in expanded skin soft tissue. Our mRNA sequencing results demonstrated that expansion significantly stimulated most DEGs in the skin, which were enriched in the biological processes of epidermal growth and keratinization, corresponding to the thickened expanded epidermis. These findings indicate significant changes in tissue remodeling and the cytoskeleton of skin tissues (Ledwon et al., 2020; Liu et al., 2021; Dong et al., 2022).

The majority of the new skin is composed of the thickened part of the epidermis, which is a result of epidermal cell growth. A transcriptomic study on expanded mouse skin reported that epidermal cells serve as crucial effector cells in skin expansion, with epidermal cell proliferation and renewal playing a decisive role in skin regeneration (Joost et al., 2018; Aragona et al., 2020). It was observed that expansion upregulates a number of HFSC-associated genes and the possible downstream differentiated cells—epidermal stem cells, including Krt14, Krt24, Cd34, Krt79, Fgf18, Lgr6, and Lgr5 (Zhang et al., 2023; Zhao et al., 2023). The epidermal basal cells of keratinocytes comprise epidermal stem cells that express Krt14 as a marker of actively proliferating keratinocytes (Wiśniewska et al., 2021). HFSCs were characterized by Krt24, Cd34, Krt79, and Fgf18 expression and were specifically located in the inner bulge cells. These cells serve as structural constituents of the epidermal cytoskeleton and contribute to skin barrier strengthening. Lgr6 encodes a member of the G protein-coupled 7-transmembrane protein superfamily. It is well known that Lgr6 is an epidermal stem cell marker, particularly during wound re-epithelialization (Huang et al., 2021). The loss of Lgr6 indicates a decrease in stemness (Ruan et al., 2019). In addition, Lgr6 activates both the Wnt and Hippo/YAP signaling pathways, which are closely related to mechanical stretching (Schlegelmilch et al., 2011). Moreover, recent studies have shown that during expansion, Lgr6+ cells in the epidermis proliferate, activate, and differentiate to stimulate skin growth (Xue et al., 2022). Lgr5 encodes a protein with high homology to the LGR6 protein (Sun et al., 2023). Both Lgr5^+^ and Lgr6^+^ cells are engaged in Wnt signaling, but Lgr5^+^ cells primarily contribute to HF renewal (Nusse and Clevers, 2017).

In addition, special transcription factors including MSX2, LEF1, TCF7, HMGA1, and TFAP2C are involved in epidermal regeneration. MSX2 encodes homeodomain transcription factors that are necessary for morphogenesis (Liu et al., 2021). It is mainly located in the epidermis and is highly influenced by stress and force, as strongly evidenced in limb regeneration. LEF1 and TCF-7 are members of the lymphoid enhancer-binding factor/T cell factor family. They drive Wnt/β-catenin signaling pathway activation (Luo et al., 2019) and play a significant role in HF differentiation. HMGA1 is highly expressed in stem cells but rarely detected in differentiated cells. By amplifying the Wnt signaling pathway, HMGA1 typically enhances stem cell self-renewal (Xian et al., 2017). TFAP2C, considered as a subunit of the transcription factor AP-2, is activated in response to stress. TFAP2C promotes the differentiation of progenitor cells into mature keratinocytes (Li et al., 2019).

Furthermore, other changes at the transcriptomic level such as circRNAs were observed to be expressed and involved in HFSC regulation (Liu et al., 2019). Collectively, all these studies analyze the changes in genes and transcription factors under mechanical stretching, which are helpful for gaining a better understanding of the underlying mechanisms. It has been identified that multiple genes and factors in HFSCs and epidermal stem cells that play a role in the biological processes promote epidermal regeneration during skin soft tissue expansion (Gentile and Garcovich, 2019).

Contrarily, the stages of HF inactivity (telogen), growth (anagen), and decline (catagen) phases depend on the complex interactions of signaling networks like Wnt/β-catenin, Sonic hedgehog (Shh), and Notch. More importantly, under the condition that HFs continually experience and are affected by intrinsic mechanical forces *in vivo*, these pathways showed directly or indirectly relationships with mechanical stretch. Consequently, this review describes the Wnt, Shh, and Notch pathways in details, advocating for further cellular studies in this field.

### 3.2 Signaling pathways in HFSCs that facilitate epidermal regeneration

#### 3.2.1 Wnt/β-catenin signaling pathway

The Wnt signaling pathway is a highly conserved cascade transduction pathway in biological processes, which participates in life activities, including embryonic development and stemness homeostasis (Zhu et al., 2014; Houschyar et al., 2020). The Wnt protein family is encoded by the *wnt* gene, which initiates complex cascade signaling reactions by binding to Frizzled membrane protein receptors and ultimately regulates the transcriptional activation of target genes. Wnt signals are transmitted through at least three different intracellular pathways, with the classical Wnt/β-catenin signal having a close relationship with HFSCs (Li et al., 2023). The Frizzled receptors are bound by the Wnt ligand, which in turn leads to the anchoring of Axin to the phosphorylated lipoprotein receptor-related protein (Ren et al., 2021). This leads to the disintegration of the complex, including Axin, APC, and GSK3, thereby resulting in β-catenin stabilization (Stamos et al., 2014; Houschyar et al., 2020). β-catenin is crucial for stabilizing the Wnt/β-catenin pathway. Notably, β-catenin acts as an effector of mechanical signals and exists as both a cytoplasmic and nuclear protein. In the plasma membrane, β-catenin can be found in two forms, the E-calmodulin/β-catenin/α-catenin complex and as unbound β-catenin (Veltri et al., 2018). β-catenin binds to E-calmodulin and α-catenin complexes through adhered junctions and participates in intercellular adhesion, migration, and cell–cell adhesion mechanotransduction when no Wnt signaling is present (Ledwon et al., 2022). When the β-catenin concentration in the cytoplasm reaches a certain level, it is translocated to the nucleus and combines with LEFs and TCF, thereby upregulating the transcription of corresponding target genes and promoting HFSC proliferation and differentiation (Nusse and Clevers, 2017).

Mechanical stimulation triggers nuclear localization of β-catenin and activates the Wnt signaling pathway, which are essential for epidermal cell skin regeneration (Cheng et al., 2020). As shown in expanded epidermis, cell thickness and density increase which are linked to WNT signaling activation and β-catenin accumulation in basal keratinocytes due to mechanical stretching (Yu et al., 2020; Guo et al., 2022). Moreover, β-catenin accumulation initiates HFSC regeneration and activation *in vivo*, followed by a formation of the positive feedback loop that continuously strengthens Wnt signaling and HFSC regeneration by transmitting mechanical stimulation to each cell through cell–matrix interactions, cell junctions, and indirect cell communication, such as non-muscle myosin II (NMM-II) protein. It is encoded by Myosin Heavy chain 9, and has been reported play an important role in responding to stiffness and rigidity sensing in cell adhesion, migration, proliferation, and differentiation. It is an important constituent of the non-muscle cytoskeleton. It is an important constituent of the non-muscle cytoskeleton (Yang et al., 2023). Research has demonstrated that MYH9 binds to GSK3, causing a decrease in GSK3β protein levels through ubiquitin-dependent degradation. The β-catenin degradation complex is impaired by this disruption, leading to the activation of the Wnt/β-catenin signaling pathway (Lin et al., 2020; Hu et al., 2022; Li et al., 2023). However, it is still worthwhile to explore the role of this protein, along with Wnt signaling and mechanical stretch.

Secreted Frizzled-related proteins and Wnt3a also regulate the activation of the Wnt/β-catenin signaling pathway. Additionally, mechanical stretching downregulates SFRP2, a Wnt pathway antagonist, thereby increasing β-catenin levels (Ledwon et al., 2022). SFRP2, in conjunction with Wnt3a or other Wnt ligands, enhances Wnt signaling activation, which is served as Wnt pathway agonists (Bai et al., 2023). Furthermore, the thickening of the expanded epidermis leads to continuous re-epithelialization in response to mechanical stimuli. New HFs gradually emerge in the central epidermis of the microtrauma areas. The growth of new HFs follows the hair cycle, resembling different stages of embryonic HF development, and ultimately becomes morphologically similar to adjacent hair (Kimura and Tsuji, 2021), suggesting the endogenous activation and recruitment of HFSCs. Research indicates that distinct stem cell compartments exist in the epidermis and HFs under physiological conditions. Upon re-epithelialization, these compartments become occupied by new epidermal cells. HFSCs temporarily differentiate into epidermal cells to cover the exposed dermis (Takeo et al., 2015). Activation of bulge cells leads to their differentiation into epidermal cells, indicating that transplanted HFSCs can become keratinocytes with enhanced proliferation capabilities, thereby facilitating epidermal regeneration. Activation of Wnt signaling in the epidermis markedly boosts the quantity of HFSCs (Rishikaysh et al., 2014). Combining other studies with our research, we are convinced that the Wnt/β-catenin signaling pathway primarily contributes to hair follicle stem cell-driven epidermal regeneration under expanded conditions.

#### 3.2.2 Sonic hedgehog (Shh) signaling pathway

The Shh pathway primarily controls signal transduction in the epidermis and can respond to mechanical loading of different tissues and stages both *in vitro* and *in vivo* (Zhang et al., 2023). HFSCs, perpetually immersed in a mechanically dynamic niche, are linked to Shh pathway activity (Huang et al., 2016). Shh signaling not only enhances both epidermal and HF growth, but preserves the stemness of HFSCs (Suen et al., 2020). Classic HH signal pathway transduction usually involves several key elements, including the HH ligand, cell surface receptor patched (PTCH), membrane protein smoothened (SMO), transcription factor glioma (GLI), and suppressor of fused protein (Zhang et al., 2023). Among the HH pathway, the Shh pathway, which is currently being extensively investigated, is essential in HFs (Ma et al., 2017). The SHH ligand binds to PTCH, thereby leading to the release of SMO, which is usually inhibited by PTCH during the resting state. The free SMO subsequently activates the target GLI.

The Shh signaling pathway plays a crucial role in HF development, primarily via enhancing dormant stem cell proliferation and modulating dermal cues that stimulate transient amplifying cell (TAC) proliferation (Hsu et al., 2014). Dormant stem cells initiate Shh expression, whereas activated stem cells give rise to TACs. The TAC population has been observed to diminish when Shh signaling is absent. Notably, the Shh pathway stimulates HF regeneration and accelerates HF maturation during skin wound healing. For example, a reduction in SHH protein selectively reduces the HF epithelium, suggesting that Shh signaling is essential for HF integrity (Lim et al., 2018). The Shh/GLI signal not only controls the development of HF in embryonic cells but also impacts the duration and growth of adult HFs by stimulating the transition of follicular cells from telogen to anagen. Furthermore, a study conducted by Choi *et al.* found that treatment with a monoclonal antibody against Shh resulted in reduced hair growth in mice (Choi, 2018). Therefore, the detailed molecular mechanisms are arousing attention and have been investigated. Mechanical stimulation influences the expression of MSX2 which is the key transcriptional regulator of follicular differentiation. Study have proposed that MSX2 can play a role in the Shh signaling pathway based on certain studies (Liu et al., 2022). When the Shh signal is activated, downstream GLI can positively regulate MSX2 expression (Anichini et al., 2021). MSX2 upregulation (Kim and Yoon, 2013) leads to an increase in LEF1 expression. Interestingly, LEF1 can also upregulate MSX2 expression in human pluripotent stem cells and other cells (Wu et al., 2015). LEF1 has also been identified as a vital transcription factor that regulates the expression of other factors involved in cytoskeleton remodeling, including matrix metalloproteinases-13 (Elayyan et al., 2017). Otherwise, LEF1, a critical component of the Wnt pathway, potentially bridges the Shh and Wnt pathways, facilitating crucial crosstalk. However, GSK3β serves as an inhibitory factor that downregulates both pathways. In addition, SFRP1, a downstream gene of the SHH pathway, has a similar effect on the Shh and Wnt pathways (Cierpikowski et al., 2023). However, the downstream GLI family of the signaling pathway can activate both of these pathways. Therefore, crosstalk between the Shh and Wnt pathways can result in antagonistic or synergistic effects in HFSCs.

#### 3.2.3 Notch signaling pathway

In the context of tissue renewal, Notch signaling plays a crucial role in regulating cell growth, directing differentiation processes, and determining cell fate. Notch signaling can be activated by mechanical stimuli under diverse conditions. Studies have shown that Notch signaling is dynamically activated and expresses in dental pulp cells, including odontoblast-like cells, in response to mechanical damage both *in vitro* and *in vivo*. Hilscher et al. have reported that the mechanical stretching of liver sinusoidal endothelial cells leads to an upsurge in CXCL1 expression. This response is mediated by the integrin-dependent triggers of transcription factors influenced by Notch, which also interact with the mechanosensitive piezo calcium channel (Hilscher et al., 2017).

The relationship between HFs and Notch pathways has been emphasized in multiple studies. A decrease in HF numbers was noted when Notch1 signaling was lacking, and the presence of Notch1-3 is noted within HF cells undergoing differentiation. Moreover, Notch signaling is crucial for preserving the integrity of the HF architecture. Previous research has indicated that during the later stages of embryonic HF development, Notch signaling is active, and mice deficient in this pathway exhibit thin, short, and curly hair (Wang et al., 2022). Moreover, elevated and active Notch1 levels have been documented in the foundational and overlying cells of sebaceous glands during preliminary epidermal layer formation (Zhu et al., 2021). Although less research has been conducted on the relationship between mechanical stretching and the Notch pathway in HFSCs, HFs continually experience and are affected by intrinsic mechanical forces *in vivo*, including pressure, compression, friction, tension, extension, shear, and mechanical injury. However, there are reasons to believe that a tight interplay exists between Notch signaling and mechanical stimuli.

### 3.3 Ion channel mechanisms

Mechanotransduction directly affects ion channels, which is primarily manifested as changes in Ca^2+^ concentration, thereby facilitating extracellular mechanical and intracellular chemical signals. Ion channels have been investigated in skin wound healing, and they are expected to play a similar role in skin expansion. Transient receptor potential (TRP) channels (Karska et al., 2023) in HFSCs are the channels involved in mechanical transduction processes. The TRP channels of the vanilloid subtype (TRPV) are mechanosensitive, and TRPV3 and TRPV4 have been identified as the main ion channels involved in HFs. Song et al. reported that HF phenotypes are affected by TRPV3 (Wang et al., 2013; Szöll˝osi et al., 2018). TRPV3 knock-in mice exhibit enhanced differentiation in HF cells due to Ca^2+^ influx activation regulation. Additionally, ClC-3 channels exhibit distinct ion control specifically related to Cl^−^. In addition, ClC-3 channel activation promotes the migration of epidermal stem cells and HFSCs to the wound area, thereby accelerating skin regeneration (Hämäläinen et al., 2021).

## 4 Conclusion and clinical perspectives

HFSCs have been proved effective in treating alopecia, inflammation, and skin wound healing through stem cell-based therapies. Although HFSCs has also been found to promote skin regeneration, especially epidermis during skin soft tissue expansion (Cheng et al., 2020), they are rarely studied in tissue expansion. They are involved in the regulation of various transcriptome changes, signaling pathways, and cellular interactions crucial for epidermal regeneration under mechanical stretch. Clinical interventions using non-invasive methods such as biological informatics, signaling pathway inhibitors/agonists, or cell therapy are beneficial for both patients and physicians. But there remain several problems which are needed to be addressed. Firstly, safety of HFSCs use is definitely the most important in clinical application. HFSCs could regulate epidermal proliferation through Wnt/β-catenin pathway which, on the other hand, leads to excessive growing of cells and results in tumor. Besides, most researches about HFSCs have been carried out on animals so far that still has some differences with human. Secondly, HFSCs are more observed in skin wound healing. Although wound healing is involved in skin expansion, the theories of HFSCs above cannot be completely appropriate in tissue expansion.

HFSCs are equipped with numerous advantages, including abundant sources, minimal harm during isolation, and versatile differentiation potential (Babakhani et al., 2020). Advances in technologies like gene knockout and transgenic techniques will enhance the use of HFSCs in skin self-renewal, tissue repair, and regeneration, broadening our understanding of HFSCs. This work will play a particularly important role in tissue engineering, regenerative medicine and other fields. Further studies should be focused on improving the effectiveness of HFSCs for skin soft tissue expansion. To fully understand the underlying mechanisms, further comprehensive investigations are necessary.
